# The Impact of a Single Haemodialysis Session on the Retinal Thickness and Optic Nerve Morphology

**DOI:** 10.3390/diagnostics14030331

**Published:** 2024-02-03

**Authors:** Joanna Roskal-Wałek, Joanna Gołębiewska, Jerzy Mackiewicz, Agnieszka Bociek, Paweł Wałek, Michał Biskup, Kamila Bołtuć-Dziugieł, Katarzyna Starzyk, Dominik Odrobina, Beata Wożakowska-Kapłon, Andrzej Jaroszyński

**Affiliations:** 1 Ophthalmology Clinic, Voivodeship Regional Hospital, 25-736 Kielce, Poland; 2 Collegium Medicum, Jan Kochanowski University, 25-516 Kielce, Poland; 3 Department of Ophthalmology, Military Institute of Aviation Medicine, 01-755 Warsaw, Poland; 4 Medical Faculty, Lazarski University, 02-662 Warsaw, Poland; 5 Department of Vitreoretinal Surgery, Medical University of Lublin, 20-079 Lublin, Poland; 6 1st Clinic of Cardiology and Electrotherapy, Swietokrzyskie Cardiology Centre, 25-736 Kielce, Poland; 7 Ophthalmology Clinic Boni Fratres Lodziensis, 93-357 Łódź, Poland

**Keywords:** retinal thickness, haemodialysis, optical coherence tomography

## Abstract

Background: The aim of the study was to assess the influence of a single haemodialysis (HD) session on the retinal and optic nerve morphology in end-stage kidney disease (ESKD) patients. Methods: It is a prospective study including only the right eye of 35 chronic kidney disease (CKD) patients subjected to HD. Each patient underwent a full eye examination 30 min before HD (8 a.m.) and 15 min after HD. Optical coherence tomography (OCT) was used to assess the peripapillary retinal nerve fibre layer (pRNFL) thickness, macular nerve fibre layer (mRNFL) thickness, ganglion cell layer with inner plexiform layer thickness (GCL+), GCL++ (mRNFL and GCL+) thickness, total retinal thickness (RT) and total macular volume (TMV). The correlation was tested between such systemic parameters changes as systolic blood pressure (SBP), diastolic blood pressure (DBP), mean arterial pressure (MAP), body weight, plasma osmolarity and ocular perfusion pressure (OPP) and ultrafiltration volume with total RT and pRNFL thickness changes during HD. Results: In the results of a single HD session, we could observe a statistically significant increase in the total RT thickness (pre-HD 270.4 ± 19.94 μm, post-HD 272.14 ± 20.11 μm; *p* = 0.0014), TMV (pre-HD 7.48 ± 0.53 mm^3^, post-HD 7.52 ± 0.55 mm^3^; *p* = 0.0006), total pRNFL thickness (pre-HD 97.46 ± 15.71 μm, post-HD 100.23 ± 14.7 μm; *p* = 0.0039), total GCL+ thickness (pre-HD 70.11 ± 9.24 μm, post-HD 70.6 ± 9.7 μm; *p* = 0.0044), and GCL++ thickness (pre-HD 97.46 ± 12.56 μm, post-HD 97.9 ± 12.94 μm; *p* = 0.0081). We observed a significant correlation between the change in total RT and DBP change, as well as between body weight change and the change in total pRNFL thickness. There was also a correlation between total pRNFL thickness change and the presence of diabetes mellitus. Conclusion: Even a single HD session affects the retinal and pRNFL thickness, which should be taken into account when interpreting the OCT results in patients subjected to HD. The impact of changes after a single HD session on selected parameters requires further assessment in subsequent studies, including long-term observation.

## 1. Introduction

Chronic kidney disease (CKD) has been a growing global health problem affecting between 8% and 16% of the population worldwide [1]. Globally, around three million patients are currently dependent on renal replacement therapies [2]. Haemodialysis (HD) is a major modality of renal replacement therapy in end-stage kidney disease (ESKD), and the frequency of its use has been increasing alongside the continuously growing incidence of CKD [2,3].

The purpose of HD is to maintain the renal excretory function in patients with ESKD. During HD, ultrafiltration removes the excess fluid from the plasma, which leads to a decrease in blood volume, increase in the plasma protein concentration, and a decrease in serum osmolarity. This volume decrease is compensated by filling the vessels with fluid from the interstitial and intercellular space [4]. Haemodynamic changes can affect retinal circulation, and these short-term changes in the retinal vessels after a single HD session may be associated with changes in RT. What is more, the changes in metabolic parameters causing osmotic alterations in aqueous and vitreous humours can also affect retinal morphology [5,6].

In studies using optical coherence tomography (OCT) in HD patients, a decrease in the retinal, choroidal and peripapillary RNFL (pRNFL) thickness was observed in HD patients compared to control groups [7,8,9,10]. Studies using the OCT-angiography (OCT-A) indicated changes in retinal microcirculation in HD patients [11]. However, it remains unclear whether the reduction in retinal and pRNFL thickness in these patients can be solely attributed to CKD or if it is influenced by the HD process itself or the frequently occurring microangiopathy associated with comorbidities such as diabetes mellitus (DM) and hypertension (HT).

The way that the retina reacts to a single HD session is not obvious, and the studies give contradictory results, from RT decrease through no changes to RT thickness increase [6,7,12,13,14,15,16,17,18,19,20,21,22,23,24,25,26]. The changes regarding pRNFL also differ in respective studies [7,13,19,24]. Only two studies assessed the reaction of selected retinal layers to HD sessions [12,13]. Changes in systemic parameters resulting from HD session and their impact on RT and pRNFL thickness changes were described only in several studies [13,14,15,16,17].

The assessment of the HD effect on the retina and optic nerve is of significant importance, especially due to the risk of decreased ocular blood flow during HD, which is also associated with the risk of ischaemic conditions within the eye and the risk of the development of glaucoma [15,27,28]. Identifying the reactions of the retina and optic nerve to an HD session is also important in the context of OCT results, as they may differ significantly depending on the time of the examination, which consequently can affect therapeutic decisions [8].

The aim of the study was to assess the influence of a single HD session on the retinal and optic nerve morphology in ESKD patients. We paid special attention to mRNFL thickness, GCL+, and GCL++ thickness, as these three layers are of key importance in the damage caused by glaucoma [29], as well as to the effect of systemic changes associated with an HD session on selected OCT parameters.

## 2. Methods

This prospective observational study was conducted between March 2022 and May 2022 at the Provincial General Hospital in Kielce. The study protocols were approved by the Bioethics Committee of the Jan Kochanowski University in Kielce (6/2022) and performed according to the provisions of the Declaration of Helsinki. Every participant provided written informed consent.

Only the right eyes of the patients were analysed. Inclusion criteria were the best corrected visual acuity (BCVA) exceeding 6/60 and no history of retinal disease except diabetic retinopathy. Exclusion criteria were spherical equivalent refractive errors more than + 3.0 D and more than −5.0 D, an axial length less than 21 mm or more than 26 mm, pathological conditions that could alter the structure of the retina or the choroid, such as macular degeneration, eye injury, intravitreal injections, laser treatment, infection, glaucoma, eye surgery, with the exception of uncomplicated cataract surgery performed at least 6 months before the study. Also, low-quality OCT images (image quality below 60, according to the manufacturer) caused by eye movements or opacification of the ocular media were excluded from the study. Other exclusion criteria were atrial fibrillation, diagnosis of acute coronary syndrome or cerebral stroke within the previous 3 months, and severe infection or exacerbation of a chronic disease within one month before the study.

All patients were subjected to three 4 h HD sessions every week with a dialysate flow rate of at least 500 mL/min and a blood flow rate of at least 300 mL/min. The patients were examined on Mondays, Wednesdays and Fridays. All the measurements were performed in the building housing the dialysis centre. In order to eliminate the impact of diurnal variations, only patients subjected to morning HD sessions were included in the study.

Each patient underwent a full eye examination 30 min before HD (8 a.m.) and 15 min after HD. All patients underwent a detailed ophthalmic examination, including a BCVA test measured on a logMAR scale, intraocular pressure (IOP) measurement, slit lamp examination of the anterior and posterior segments, and OCT of the macula and optic nerve. All scans were obtained using swept-source OCT-A (DRI-OCT Triton SS-OCT Angio, Topcon Inc., Tokyo, Japan).

The OCT protocols included 3D macula 7 × 7 mm scanning protocols and 3D 6 × 6 mm disc scanning protocols. Retinal thickness measurement was performed automatically in nine subfields of the ETDRS grid using a 7 × 7 mm 3D scan of the macula. The analysed retinal layers and parameters included RT, macular RNFL (mRNFL) thickness, GCL+ (ganglion cell layer with inner plexiform layer thickness), and GCL++ (mRNFL and GCL+) thickness and total macular volume (TMV). Total RT, as well as total mRFNL thickness, total GCL+ thickness and total GCL++ thickness, were the average thickness of nine subfields of the ETDRS grid for each layer. Peripapillary RNFL thickness was evaluated in each of the four quadrants using a radial scan centred on the optic nerve head. We analysed the following parameters: Total pRNFL—average thickness for 360°; superior pRNFL—thickness in the superior 90°, inferior pRNFL—thickness in the inferior 90°; nasal pRNFL—thickness in the nasal 90°; and temporal pRNFL—thickness in the temporal 90°.

Patients with ESKD were divided into two groups: patients with diabetes mellitus (DM) and patients without diabetes mellitus (NDM). None of the DM patients had diabetic retinopathy (DR).

The body weight, plasma osmolarity, systolic blood pressure (SBP), diastolic blood pressure (DBP) and mean arterial pressure (MAP) were measured before and after HD. MAP was calculated using the formula DBP + 1/3 (SBP–DBP). The ultrafiltration volume represented the amount of fluid removed during HD and was recorded after HD. Additionally, ocular perfusion pressure (OPP) was calculated using the formula 2/3 MAP–IOP.

## 3. Statistical Analysis

The Wilcoxon signed-rank test was used to compare measurements of the entire study group at two time points—before and after HD. Subgroup analysis comparing patients with DM and without DM was performed using the Mann–Whitney U test. Correlations between measurements were calculated using Spearman’s correlation test. For single factors, the simple regression model was used. The threshold of statistical significance was set at *p* < 0.05. Statistical analyses were performed with STATISTICA 13 software (TIBCO Software Inc., Tulsa, OK, USA)

## 4. Results

### 4.1. Characteristics of the Study Group

A total of 48 patients were examined, of which 35 patients met the inclusion criteria, and 35 eyes were analysed in the study: 18 women and 17 men. The mean age was 61.14 ± 12.01 (32–83) years. Among the included patients the cause of ESKD were DM (*n* = 8), glomerulonephritis (*n* = 7), hypertension (*n* = 6), polycystic kidney disease (*n* = 4), vasculitis (*n* = 2), other (*n* = 3), unknown (*n* = 5). The mean duration of HD was 4.85 ± 3.65 years. The baseline characteristics of the group are presented in Table 1.

### 4.2. Effect of Haemodialysis on Systemic Parameters

The body weight changed during HD from 77.83 ± 18.64 to 75.64 ± 18.21 kg (*p* < 0.0001).

The plasma osmolarity changed during HD from 321.12 ± 9.81 to 302.79 ± 7.66 mOsm/kg (*p* < 0.0001). The mean change in brachial diastolic, systolic or mean arterial pressure was not significant (*p* > 0.05). The detailed changes of the above-mentioned parameter are presented in Table 2.

### 4.3. Effect of Haemodialysis on BCVA, IOP and OPP

The mean logMAR BCVA significantly improve after HD (pre-HD 0.15 ± 0.14, post-HD 0.12 ± 0.16; *p* = 0.0096). The mean change in IOP and OPP was not significant (*p* > 0.05). Table 3 summarises the effects of haemodialysis on ocular parameters.

### 4.4. Effect of Haemodialysis on TMV, Total RT and pRNFL Thickness

The mean total RT and TMV significantly increase after HD. The mean pRNFL increased significantly in superior, nasal, and inferior subfields, including the total (*p* < 0.05). The detailed changes of the above-mentioned parameter are presented in Table 4.

### 4.5. Effect of Haemodialysis on Separate Retinal Layers

No changes were observed in mRNFL thickness, either in the total or in respective subfields (*p* > 0.05). In the case of GCL+ and GCL++, the measured thickness increased in the inner and outer subfields, as well as in the total (*p* < 0.05). In these layers, there were no changes observed in the central subfield (*p* > 0.05). The above-mentioned changes are summarised in Table 5.

### 4.6. Effect of Haemodialysis on TMV, RT and pRNFL Thickness in Subgroups of Patients with and without DM

In both the NDM and DM groups, the RT increase was observed in most of the subfields, including the total (*p* < 0.05). Also, in the entire group, the RT increase in most of the subfields, including the total, was demonstrated (*p* < 0.05). A detailed analysis of the effect of HD on RT is presented in Table 6.

### 4.7. Comparison of RT and pRNFL Thickness between Subgroups of Patients with and without DM

A significant post-HD difference between NDM and DM patients was observed only in the inner superior and inner nasal subfields of RT, as well as in the nasal and temporal subfields of the pRNFL (*p* < 0.05). The comparison of RT between NDM and DM patients is presented in Table 7.

### 4.8. Analysis of Correlations between Changes in Systemic Parameters and Changes in RT and pRNFL Thickness

Correlations between systemic parameters and RT or pRNFL (*p* < 0.05) were observed—Table 8. The change of RT correlated with the change of DBP (R = −0.397), while the change of pRNFL correlated with ultrafiltration volume (R = −0.364) and DM (R = −0.355).

Simple (single factor) regression analysis was performed to determine dependencies between RT and pRNFL changes and systemic measurement changes. There were no strong correlations between changes in RT or pRNFL thickness and changes in any other analysed parameter (Eta^2^ < 0.15, R^2^ < 0.15).

## 5. Discussion

In this study, we found that there was a statistically significant increase in total RT, TMV and total pRNFL thickness after a single HD session for the entire study group. In selected layers of the retina, we observed a statistically significant increase in total GCL+ and GCL++, but we did not observe an increase in total mRNFL thickness. In both the NDM and DM groups, the RT increase in most of the subfields, including the total, was observed. The significant difference between DM and NDM patients was only observed in the inner superior and inner nasal subfields of RT and the nasal and temporal subfields of pRNFL after HD. The change in RT correlated with the change in DBP, and the change in pRNFL thickness correlated with the change in body weight. There was also a correlation between pRNFL change and the presence of DM.

The results of our study regarding the increase in RT as a result of a single HD session are consistent with the results of Wang et al., Atilgan et al., and Jabbar et al. [7,15,24]. In this study, we also found a statistically significant increase in total pRNFL thickness, which is in agreement with previous studies by Atilgan et al. and Jabbar et al. in which a statistically significant increase in pRNFL thickness was found after HD session [7,24].

However, a significant number of studies indicate no effect of an HD session on RT or even a decrease in RT after HD [6,12,13,16,17,18,19,20,21,22,23,25,26]. The differences may be due to small groups of subjects, methodological differences between studies, differences in the OCT types used, and differences in the groups of subjects themselves, including age, gender, axial length, refraction, or ethnic origin, all of which influence the retinal, GCL and pRNFL thickness [6,7,12,13,14,15,16,17,18,19,20,21,22,23,24,25,26,30]. The aetiology of CKD in the studied patients subjected to HD may be of particular importance with respect to differences in the obtained results. The impact of DM and the associated microangiopathy may affect not only the baseline RT but also its response to HD. The available literature includes studies assessing RT in the NDM group only [7,15,18,25], mixed groups of DM and NDM patients [6,12,13,14,16,17,19,21,22,23,26] or DM patients who were also assessed with respect to the degree of DR or the presence of macular oedema [20,24,31].

In the study by Sun et al., no statistically significant change in the central macular thickness (CMT) was observed following an HD session in the entire study group, as well as various aetiological groups, including chronic glomerulonephritis, hypertension and other causes. The CMT changes in the study by Sun et al. were statistically significant only in the case of DM patients, in whom CMT reduction was observed following HD [16]. In the study by Chen et al., RT increased after HD, especially in the nasal inner macula subfield (*p* < 0.001), the inferior inner macula subfield (*p* = 0.004) and the superior outer macula subfield (*p* = 0.012). However, average thickness and central subfield thickness did not demonstrate statistically significant changes in the entire study group. An increase in RT was present in primary kidney disease, hypertension (HT) kidney disease and DM kidney disease groups. In this study, although RNFL increased in the entire group, the thickness in DM patients before and after HD was lower, and a lower change in RNFL thickness was found in the DM patients compared to the other two subgroups [14]. However, in the study by Chelala et al., patients with different CKD aetiologies, such as DM and HT, did not differ in terms of CMT response to HD. CMT in all the groups analysed in this study did not demonstrate statistically significant differences [6]. Additionally, in the study by Yang et al., no statistically significant changes were found in central foveal thickness (CFT), macular volume (MV) or pRNFL thickness in the entire study group, which also included patients with DM. The authors attributed these results to the autoregulation system in the retina, which is not as highly vascularised as the choroid. It is worth mentioning that the autoregulatory capacity of the retina may be impaired due to the presence of microangiopathy, which may result, for example, from the presence of DM [19]. Furthermore, it should be kept in mind that in HD patients with CKD, episodes of intradialytic hypotension and hypertension may also cause endothelial dysfunction and an increase in arterial stiffness. Moreover, CKD often involves increased inflammation and oxidative stress, dysregulation of the renin-angiotensin system and the presence of uraemic toxins, which may exacerbate neuroinflammation, increase free radical formation, and cause vascular dysfunction [32]. HD itself also increases inflammation state [33].

In the study by Yang et al., the change in CFT after HD was different in patients with DM compared to NDM patients. The mean CFT in patients with DM decreased from 218.2 ± 34.7 to 214.6 ± 35.8 μm, while the mean CFT in NDM patients increased from 211.7 ± 14.1 to 213.1 ± 14.5 μm after HD. These differences between both groups were statistically significant (*p* = 0.003). Mean MV was slightly decreased in the DM group and increased in the NDM group after HD, but these changes were not statistically significantly different. Changes in total pRNFL thickness did not differ significantly between the DM and NDM groups. The authors noted that the blood–retinal barrier (BRB) might play a role in changes in CFT and MV in DM and NDM patients after HD, but the mechanism is unknown [19].

In our group, we observed a statistically significant increase in total RT in DM and NDM patients. The comparison of RT and pRNFL thickness before and after HD in the respective groups demonstrated a significant difference between non-DM and DM patients only in the inner superior and inner nasal subfields of RT and nasal and temporal subfields of pRNFL after HD. However, our DM group was relatively small, and none of these patients had DR. That is why these results should be interpreted with caution.

In a study by Wang et al., including NDM patients, an increase in RT was also observed. The authors noted that the retina, being a nervous tissue with complex self-regulatory functions, may be susceptible to retinal oedema after HD [15].

Chen et al. assumed that HD reduces the plasma crystal osmotic pressure in such a way that the liquid flows into the retinal layers according to the concentration gradient, thickening the retina and leading to oedema [14]. We believe that this mechanism may be responsible for similar changes observed in our study. This explanation is very interesting, especially in relation to the changes that occur in the central nervous system (CNS) during HD, and it is justified, especially considering that, anatomically and developmentally, the retina is known to be an extension of the CNS [34]. HD may induce osmolar and fluid shifts, increasing brain water content with the potential for cerebral oedema. The development of cerebral oedema can lead to dialysis disequilibrium syndrome, a rare but serious neurological complication of HD [35]. If the observed increase in retinal and pRNFL thickness is analogous to the changes occurring in the CNS, it may indicate that swelling of the nervous tissue occurs during HD, but its degree may vary individually. Thus, it may lead to the occurrence of symptoms only in some cases. However, this hypothesis requires verification in subsequent studies.

It is worth noting that changes in the aquaporins (AQP) channels may play an important role in the development of cerebral oedema. Aquaporins are small cell membrane proteins that, in the presence of an osmotic gradient, allow water and sometimes small molecules, such as carbon dioxide and ammonia, to pass through. AQPs are present in abundance in the RPE, inner nuclear layer, and ganglion cell layer, while they are absent in the RNFL layer [34]. Perhaps the type of AQP and the degree of its expression in different layers are related to the increase in GCL thickness observed in our study, but this requires evaluation in subsequent studies.

It has been indicated that haemodynamic changes occurring during HD may significantly affect blood flow in the retina, which may result in RT alteration [5,6,16]. In the study by Sun et al., retinal arterial calibre and retinal venous calibre were assessed, and it was found that they increased significantly after HD. Dilation of retinal microcirculation vessels after HD may result from the release of vasoactive factors, such as nitric oxide, the availability of which increases as a result of the removal of nitric oxide synthase inhibitor- asymmetric dimethylarginine during HD. However, despite these changes observed in the study by Sun et al., CMT did not change significantly in the entire group after a single HD session [16].

On the other hand, as the authors of other studies pointed out, vasodilation is masked as a result of increased vascular resistance and increased haematocrit and hypotension after HD. Therefore, retinal blood flow does not change [5,7].

What happens as a result of HD in microvessels is better demonstrated by studies using OCT-A, which allows for a precise assessment of changes in the flow in the retinal vascular plexuses and choriocapillaris. In the study by Zhang et al., there was a decrease in vessel density (VD) in the outer retina, which was consistent with a decrease in RT, and no changes were observed in the superficial capillary plexus (SCP), deep capillary plexus (DCP) and choriocapillaris VD [22]. According to Shin et al., there were also no changes in SCP and DCP-VD, but there was a visible decrease in choriocapillaris VD, while the RT did not change [21]. In the study by Coppolino et al., a statistically significant decrease for 6 × 6 whole VD of SCP and 6 × 6 foveal VD of DCP were observed after HD in the entire group; however, there was no statistically significant change in RT [36].

The impact of systemic changes that occur during HD, which vary in different studies, may lead to the final difference in the obtained results regarding both RT and pRNFL thickness [12,13,14,15,16,17]. In our study, there was a statistically significant correlation between the change in body weight and the change in pRNFL thickness, as well as between the change in DBP and the change in RT. However, this correlation was very weak and requires verification in subsequent studies. Most studies analysing the effect of systemic parameters on RT did not demonstrate a significant correlation between changes in the assessed parameters and changes in RT [15,16,17,23]. Chen et al. found a positive correlation between the mean change in RT and potassium levels before HD [14]. In the study by Emre et al., the change in mean arterial pressure (MAP) was statistically significant, but the decrease in MAP did not correlate with the central fovea, ganglion cell layer, central subfield or RNFL thickness [13].

Frequently reported in HD patients, a decrease in MAP and an increase in IOP may lead to a decrease in ocular perfusion pressure (OPP) and consequently may result in the occurrence of ischaemic changes, including the risk of developing open-angle glaucoma [28].

In our study, we did not observe statistically significant changes in IOP, OPP, or any correlation between OPP changes and changes in RT or pRNFL following HD. In the assessment of damage associated with glaucoma, GCL plays a particularly important role, and therefore, we decided to also assess these layers in our study. We found that GCL+ and GCL++ thickened, which was not reflected in the mRNFL. Maharshak et al. assessed the impact of an HD session on the layers of the retina and did not demonstrate statistically significant changes in the thickness of the retinal layers after an HD session, which led to the conclusion that HD does not have a negative effect on the retina [12]. In the study by Emre et al., in the entire group after an HD session, a reduction in the central foveal thickness and GCL thickness was observed, while the central subfield and RNFL thickness increased, but the values did not reach statistical significance [13].

It is worth noting that repeated changes in parameters related to retinal and pRNFL thickness caused by HD may have a long-term effect [7,20]. In the study by Atilgan et al., the RNFL and macular thickness before and after HD in NDM patients were assessed on the first day, after one month and then after six months. Superior and average RNFL thickness values after HD turned out to be significantly higher than before HD on the first day and after the first month (*p* < 0.05). This increase in thickness was only observed in the superior quadrant at month six (*p* = 0.048). Macular thickness values in all quadrants, except the mean value, were significantly higher on the first day after HD than before HD (*p* < 0.05). This increase was found to be significant in the superior, nasal and temporal quadrants after the first month (*p* < 0.05). There was no significant difference between macular thickness values before and after HD after the sixth month (*p* > 0.05). The only consistent effect of HD was found to be an increase in superior quadrant RNFL thickness [7].

As indicated above, the currently available study results are very diverse, and the reasons for the reduction, lack of reaction or increase in RT and pRNFL thickness following HD observed in previous studies are not clear [6,7,12,13,14,15,16,17,18,19,20,21,22,23,24,25,26,36]. There is no doubt that further research is needed, including the use of OCT-A, which will allow us to address changes in microcirculation and their potential impact on RT. It should also be noted that the retina is vascularised in 2/3 by the central retinal artery and in 1/3 by the choroidal circulation; therefore, changes in the choroidal thickness during HD may also affect the RT thickness [22,37,38]. In addition, the blood supply to the optic nerve and optic nerve head remains under the influence of the nervous system. Blood flow in the retinal vessels themselves is controlled at the level of the central retinal artery, short ciliary arteries and the choroid, as well as by local retinal metabolic regulatory mechanisms and neurovascular coupling [37]. In the context of the thinning of the retina and choroid observed in patients with CKD, attention is also drawn to disturbances in the functions of the autonomic system, which, depending on the degree of damage, may influence the observed changes in the morphology of the retina and choroid, also in response to a single HD session [4,38]. Moreover, a number of changes in ocular parameters that occur during HD may be interconnected and be a part of the overall response of the eye to HD-related stress, which has not been known yet.

## 6. Limitations

This study was conducted on a relatively small group, and the assessment of changes in selected parameters concerned only the impact of a single HD session. Another limitation was the lack of a follow-up, as we did not carry out further measurements after subsequent HD sessions to observe whether these changes were repeatable and what changes in RT and pRNFL thickness they caused in a specific time period. In this study, we did not present the correlation with the OCT-A results, nor did we present the effect of HD on choroidal thickness.

We did not consider the influence of drugs, changes in gas concentration, pH and inflammatory factors, or changes in Na, K and Ca concentration on the assessed parameters.

Regarding the comparison of DM and NDM patients, in the DM group, there were no patients with DR, which did not provide us with insight into the retinal response in patients with DM and concomitant retinopathy. On the other hand, the evaluation of DM patients without DR may provide information on whether there are already visible OCT changes in retinal and pRNFL thickness after a single session of HD in DM patients before the development of DR.

## 7. Strengths

In this study, we used automatic measurement, which excluded the presence of measurement error associated with manual measurement. We assessed not only RT but also took into account the impact of HD sessions on RNFL and GCL, the changes of which have an important role in the development of glaucoma. We addressed numerous systemic parameters in order to better assess the variables that may be correlated with the change in RT and pRNFL thickness as a result of a single HD session. The assessment of the impact of an HD session on selected parameters was performed immediately before and after HD, which significantly reduced the impact of time and other independent factors on the obtained results.

## 8. Conclusions

Our study indicates that a single HD session has a significant effect on the retinal and pRNFL morphology. Whether these changes are repeatable and what long-term consequences they may lead to requires further research. It remains unclear whether the thickening of RT and pRNFL results from changes that can be classified as those related to the process of autoregulation to the ongoing systemic changes or whether tissue oedema is responsible, or a vascular component is involved. The results of our study indicate that a single HD session affects the obtained measurements of retinal and pRNFL thickness, and the differences in the measured values are statistically significant, indicating that the time at which OCT examinations are performed in HD patients should be taken into account. This is particularly important in assessing patients for the presence of changes associated with glaucoma or in monitoring the existing changes. Moreover, when comparing the examination results, it is necessary to consider the interval between the HD session and the OCT examination.

## Figures and Tables

**Table 1:** Baseline characteristics of the study group.

Patients’ Characteristic	Summary Statistics
Age (years)	61.14 ± 12.01
Mean (SD)	
Gender	
Females (%)	18 (51.43%)
Males (%)	17 (48.57%)
Duration of HD (years)	4.85 ± 3.65
Mean (SD)	
Ultrafiltration volume (l)	2.19 ± 1.03
Mean (SD)	
kt/v	1.6 ± 0.31
Mean (SD)	
Spherical equivalent, (D)	0.15 ± 1.55
Mean (SD)	
Axial length, (mm)	22.94 ± 0.79
Mean (SD)	

Abbreviations: HD, haemodialysis; SD, standard deviation.

**Table 2:** Effect of haemodialysis on systemic parameters.

	Before HD	After HD	*p*-Value
Body weight (kg)	77.83 ± 18.64	75.64 ± 18.21	<0.0001
SBP (mmHg)	141.54 ± 27.63	137.65 ± 28.66	0.4638
DBP (mmHg)	77.63 ± 13.7	78.41 ± 14.18	0.7154
MAP (mmHg)	98.93 ± 17.42	98.16 ± 18.09	0.9715
Plasma osmolarity (mOsm/kg)	321.12 ± 9.81	302.79 ± 7.66	<0.0001

Abbreviations: DBP, diastolic blood pressure; HD, haemodialysis; MAP, mean arterial pressure; SBP, systolic blood pressure.

**Table 3:** Effect of haemodialysis on BCVA, IOP and OPP.

	Before HD	After HD	*p*-Value
BCVA, (logMAR)	0.15 ± 0.14	0.12 ± 0.16	0.0096
IOP (mmHg)	14.94 ± 3.68	14.74 ± 3.76	0.3571
OPP (mmHg)	76.92 ± 15.4	74.9 ± 17.24	0.1549

Abbreviations: BCVA, best-corrected visual acuity; HD, haemodialysis; IOP, intraocular pressure; OPP, ocular perfusion pressure.

**Table 4:** Effect of haemodialysis on TMV, total RT and pRNFL thickness.

		Before HD	After HD	*p*-Value
Total RT (μm)		270.4 ± 19.94	272.14 ± 20.11	0.0014
TMV (mm^3^)		7.48 ± 0.53	7.52 ± 0.55	0.0006
Total pRNFL thickness (μm)		97.46 ± 15.71	100.23 ± 14.7	0.0039
pRNFL thickness (μm)	Superior	118.2 ± 22.91	123.03 ± 23.51	0.0023
	Temporal	72.03 ± 14.13	71.31 ± 10.29	0.2264
	Nasal	76.77 ± 13.66	80.46 ± 12.13	0.0499
	Inferior	121.54 ± 27.66	126.63 ± 23.6	0.0125

Abbreviations: HD, haemodialysis; pRNFL, peripapillary retinal nerve fibre layer; RT, retinal thickness; TMV, total macular volume.

**Table 5:** Effect of haemodialysis on separate retinal layers.

		Before HD	After HD	*p*-Value
mRNFL (μm)	C	3.31 ± 2.27	3.31 ± 2.71	0.4096
	OS	38.18 ± 6.4	38.39 ± 6.52	0.1802
	OI	38.7 ± 8.61	38.21 ± 8.31	0.0738
	ON	45.27 ± 8.86	45.58 ± 9.13	0.0723
	OT	20.58 ± 5.33	20.42 ± 5.17	0.4051
	IS	27.37 ± 2.65	26.83 ± 3.33	0.0711
	II	28.23 ± 3.6	28.2 ± 3.63	0.4355
	IN	23.17 ± 1.95	23.13 ± 2.34	0.4588
	IT	18.23 ± 4.97	18.37 ± 4.6	0.4294
	To	27.34 ± 3.91	27.26 ± 3.91	0.4412
GCL+ (μm)	C	43.76 ± 7.75	43.36 ± 8.18	0.0743
	OS	60.64 ± 6.69	61.33 ± 6.99	0.0172
	OI	59.67 ± 7.44	60.79 ± 8.93	0.0041
	ON	64.85 ± 8.22	65.94 ± 8.64	0.0001
	OT	66.03 ± 7.53	66.61 ± 7.86	0.0502
	IS	84.18 ± 14.14	84.58 ± 14.25	0.0269
	II	85.06 ± 13.92	85.97 ± 14.29	0.0003
	IN	85.58 ± 12.5	86.06 ± 12.43	0.0129
	IT	82.97 ± 12.82	83.27 ± 13.43	0.1497
	To	70.11 ± 9.24	70.6 ± 9.7	0.0044
GCL++ (μm)	C	47.15 ± 9.25	47.06 ± 9.52	0.4276
	OS	98.58 ± 11.64	99.61 ± 12.15	0.0005
	OI	100.15 ± 12.55	100.82 ± 13.24	0.0287
	ON	112.36 ± 13.9	113.24 ± 14.53	0.0019
	OT	86.88 ± 11.1	87.21 ± 11.32	0.0569
	IS	111.48 ± 16.04	111.52 ± 16.43	0.0764
	II	113.48 ± 16.93	114.18 ± 16.93	0.0111
	IN	108.76 ± 13.68	109.24 ± 13.72	0.0267
	IT	101.39 ± 16.51	101.91 ± 16.67	0.0121
	To	97.46 ± 12.56	97.9 ± 12.94	0.0081

Abbreviations: C, central; GCL +, ganglion cell layer with inner plexiform layer thickness; GCL++ (mRNFL and GCL+); HD, haemodialysis; II, inner inferior; IN, inner nasal; IS, inner superior; IT, inner temporal; OI, outer inferior; ON, outer nasal; OS, outer superior; OT, outer temporal; mRNFL, macular retinal nerve fibre layer; To, total.

**Table 6:** Effect of haemodialysis on TMV, RT and pRNFL thickness in subgroups of patients with and without DM.

Structure/Subfield		Non-Diabetes Mellitus (*n* = 27)		*p*-Value	Diabetes Mellitus (*n* = 8)		*p*-Value	Overall (*n* = 35)		*p*-Value
		Before HD	After HD		Before HD	After HD		Before HD	After HD	
RT (μm)	C	239.19 ± 25.52	240.78 ± 26.27	0.0255	220.88 ± 29.26	228.13 ± 28.96	0.009	235 ± 27.11	237.89 ± 27.01	0.0024
	OS	260.59 ± 15.14	262.15 ± 15.88	0.014	248.25 ± 23.58	249.88 ± 23.75	0.026	257.77 ± 17.82	259.34 ± 18.34	0.0027
	OI	256.74 ± 17.89	257.37 ± 18.18	0.2669	243.38 ± 22.6	245.38 ± 23.31	0.0216	253.69 ± 19.55	254.63 ± 19.76	0.116
	ON	273.67 ± 18.72	275.56 ± 19.31	0.0018	260.13 ± 28.33	263.5 ± 28.17	0.0059	270.57 ± 21.6	272.8 ± 21.79	0.0001
	OT	249.56 ± 13.73	250 ± 14.01	0.1803	235.88 ± 30.13	238.25 ± 31.62	0.1726	246.43 ± 19.11	247.31 ± 19.52	0.0954
	IS	298.89 ± 15.58	297.89 ± 22.4	0.0914	277.25 ± 36.73	278.63 ± 36.04	0.0881	293.94 ± 23.42	293.49 ± 26.81	0.0378
	II	297.41 ± 21.03	301.78 ± 16.73	0.0014	280.75 ± 40.43	282.63 ± 40.22	0.025	293.6 ± 26.93	297.4 ± 24.77	0.0002
	IN	302.07 ± 17.65	304.11 ± 18.75	0.0057	280.25 ± 36.25	283.75 ± 30.23	0.04	297.09 ± 24.4	299.46 ± 23.07	0.0014
	IT	289.85 ± 13.04	291.19 ± 13.67	0.0156	271 ± 43.65	272.63 ± 42.98	0.0881	285.54 ± 24.22	286.94 ± 24.2	0.0061
	To	274.22 ± 14.17	275.65 ± 15.14	0.0102	257.53 ± 30.64	260.31 ± 30.11	0.025	270.4 ± 19.94	272.14 ± 20.11	0.0014
pRNFL thickness (μm)	S	117.93 ± 23.97	123.19 ± 23.38	0.0011	119.13 ± 20.38	122.5 ± 25.56	0.3897	118.2 ± 22.91	123.03 ± 23.51	0.0023
	I	122.63 ± 28.92	128.7 ± 23.29	0.0255	117.88 ± 24.29	119.63 ± 24.88	0.2004	121.54 ± 27.66	126.63 ± 23.6	0.0125
	N	76.26 ± 14.53	79.96 ± 12.59	0.1104	78.5 ± 10.89	82.13 ± 11.05	0.1184	76.77 ± 13.66	80.46 ± 12.13	0.0499
	T	72.74 ± 14.6	72.22 ± 9.6	0.1208	69.63 ± 13.06	68.25 ± 12.59	0.2771	72.03 ± 14.13	71.31 ± 10.29	0.2264
	To	97.41 ± 16.08	101 ± 14.46	0.0007	97.63 ± 15.4	97.63 ± 16.19	0.2643	97.46 ± 15.71	100.23 ± 14.7	0.0039
TMV (mm^3^)		7.58 ± 0.41	7.61 ± 0.43	0.004	7.15 ± 0.79	7.22 ± 0.8	0.025	7.48 ± 0.53	7.52 ± 0.55	0.0006

Abbreviations: C, central; DM, diabetes mellitus; HD, haemodialysis; I, inferior; II, inner inferior; IN, inner nasal; IS, inner superior; IT, inner temporal; N, nasal; OI, outer inferior; ON, outer nasal; OS, outer superior; OT, outer temporal; pRNFL, peripapillary retinal nerve fibre layer; RT, retinal thickness; S, superior; T, temporal; To, total; TMV, total macular volume.

**Table 7:** Comparison of RT and pRNFL thicknessbetween subgroups of patients with and without DM.

Structure/Subfield		Before HD		*p*-Value	After HD		*p*-Value
		NDM (*n* = 27)	DM (*n* = 8)		NDM (*n* = 27)	DM (*n* = 8)	
RT (μm)	C	239.19 ± 25.52	220.88 ± 29.26	0.0772	240.78 ± 26.27	228.13 ± 28.96	0.2141
	OS	260.59 ± 15.14	248.25 ± 23.58	0.0715	262.15 ± 15.88	249.88 ± 23.75	0.0662
	OI	256.74 ± 17.89	243.38 ± 22.6	0.0832	257.37 ± 18.18	245.38 ± 23.31	0.1431
	ON	273.67 ± 18.72	260.13 ± 28.33	0.1431	275.56 ± 19.31	263.5 ± 28.17	0.1816
	OT	249.56 ± 13.73	235.88 ± 30.13	0.1431	250 ± 14.01	238.25 ± 31.62	0.2375
	IS	298.89 ± 15.58	277.25 ± 36.73	0.0335	297.89 ± 22.4	278.63 ± 36.04	0.0335
	II	297.41 ± 21.03	280.75 ± 40.43	0.0961	301.78 ± 16.73	282.63 ± 40.22	0.0715
	IN	302.07 ± 17.65	280.25 ± 36.25	0.0306	304.11 ± 18.75	283.75 ± 30.23	0.0207
	IT	289.85 ± 13.04	271 ± 43.65	0.1816	291.19 ± 13.67	272.63 ± 42.98	0.1616
	To	274.22 ± 14.17	257.53 ± 30.64	0.0715	275.65 ± 15.14	260.31 ± 30.11	0.118
pRNFL thickness (μm)	S	117.93 ± 23.97	119.13 ± 20.38	0.2621	123.19 ± 23.38	122.5 ± 25.56	0.4162
	I	122.63 ± 28.92	97.63 ± 15.4	0.1616	128.7 ± 23.29	119.63 ± 24.88	0.118
	N	76.26 ± 14.53	78.5 ± 10.89	0.0715	79.96 ± 12.59	82.13 ± 11.05	0.0207
	T	72.74 ± 14.6	69.63 ± 13.06	0.1344	72.22 ± 9.6	68.25 ± 12.59	0.0335
	To	97.41 ± 16.08	97.63 ± 15.4	0.2029	101 ± 14.46	97.63 ± 16.19	0.4012

Abbreviations: C, central; DM, diabetes mellitus; HD, haemodialysis; I, inferior; II, inner inferior; IN, inner nasal; IS, inner superior; IT, inner temporal; N, nasal; NDM, non-diabetes mellitus; OI, outer inferior; ON, outer nasal; OS, outer superior; OT, outer temporal; pRNFL, peripapillary retinal nerve fibre layer; RT, retinal thickness; S, superior; T, temporal; To, total; TMV, total macular volume.

**Table 8:** Correlations between changes in systemic parameters and changes in RT and pRNFL thickness (significant correlations were **bolded**; *p* < 0.05).

	Delta Total RT	Delta Total pRNFL
Age	0.037	0.02
Sex	0.112	−0.249
DM	0.155	**−0.355**
Duration of HD	−0.219	−0.214
Ultrafiltration volume	−0.229	**−0.364**
Delta SBP	−0.215	−0.009
Delta DBP	**−0.397**	0.053
Delta MAP	−0.307	−0.004
Delta plasma osmolarity	−0.24	0.183
Delta mOPP	−0.158	0.193

Abbreviations: DBP, diastolic blood pressure; DM, diabetes mellitus; HD, haemodialysis; MAP, mean arterial pressure; mOPP, mean ocular perfusion pressure; pRNFL, peripapillary retinal nerve fibre layer; RT, retinal thickness; SBP, systolic blood pressure.

## Data Availability

Data are contained within the article.
